# APESTNet with Mask R-CNN for Liver Tumor Segmentation and Classification

**DOI:** 10.3390/cancers15020330

**Published:** 2023-01-04

**Authors:** Prabhu Kavin Balasubramanian, Wen-Cheng Lai, Gan Hong Seng, Kavitha C, Jeeva Selvaraj

**Affiliations:** 1 Department of Data Science and Business System, Kattankulathur Campus, SRM Institute of Science and Technology, Chennai 603203, Tamil Nadu, India; 2 Bachelor Program in Industrial Projects, National Yunlin University of Science and Technology, Douliu 640301, Taiwan; 3 Department of Electronic Engineering, National Yunlin University of Science and Technology, Douliu 640301, Taiwan; 4 Department of Data Science, UMK City Campus, University Malaysia Kelantan, Pengkalan Chepa, Kelantan 16100, Malaysia; 5 Department of Computer Science and Engineering, Sathyabama Institute of Science and Technology, Chennai 600119, Tamil Nadu, India

**Keywords:** adversarial propagation, liver tumor segmentation, classification, enhanced swin transformer network, median filtering, computed tomography

## Abstract

**Simple Summary:**

The classification is performed later by an interactively learning Swin Transformer block, the core unit for feature representation and long-range semantic information. In particular, the proposed strategy improved significantly and was very resilient while dealing with small liver pieces, discontinuous liver regions, and fuzzy liver boundaries. The experimental results confirm that the proposed APESTNet is more effective in classifying liver tumours than the current state-of-the-art models. Without compromising accuracy, the proposed method conserved resources. However, the proposed method is prone to slight over-segmentation or under-segmentation errors when dealing with lesions or tumours at the liver boundary. Therefore, our future work will concentrate on completely utilizing the z-axis information in 3D to reduce errors.

**Abstract:**

Diagnosis and treatment of hepatocellular carcinoma or metastases rely heavily on accurate segmentation and classification of liver tumours. However, due to the liver tumor’s hazy borders and wide range of possible shapes, sizes, and positions, accurate and automatic tumour segmentation and classification remains a difficult challenge. With the advancement of computing, new models in artificial intelligence have evolved. Following its success in Natural language processing (NLP), the transformer paradigm has been adopted by the computer vision (CV) community of the NLP. While there are already accepted approaches to classifying the liver, especially in clinical settings, there is room for advancement in terms of their precision. This paper makes an effort to apply a novel model for segmenting and classifying liver tumours built on deep learning. In order to accomplish this, the created model follows a three-stage procedure consisting of (a) pre-processing, (b) liver segmentation, and (c) classification. In the first phase, the collected Computed Tomography (CT) images undergo three stages of pre-processing, including contrast improvement via histogram equalization and noise reduction via the median filter. Next, an enhanced mask region-based convolutional neural networks (Mask R-CNN) model is used to separate the liver from the CT abdominal image. To prevent overfitting, the segmented picture is fed onto an Enhanced Swin Transformer Network with Adversarial Propagation (APESTNet). The experimental results prove the superior performance of the proposed perfect on a wide variety of CT images, as well as its efficiency and low sensitivity to noise.

## 1. Introduction

The liver provides essential support for animals and vertebrates on this planet. Liver disease is a potentially fatal condition with no warning signs in the human body. The patient’s prognosis would greatly benefit from an early diagnosis of liver illness. The incidence of liver tumours is high, making it one of the most lethal forms of cancer. Radiologists face major challenges in the early analysis and accurate staging of liver cancer. The United States reports that liver cancer ranks as the tenth foremost cause of cancer overall, the fifth foremost cause of cancer death in men, and the ninth leading motive of cancer [1,2]. When cancer is identified at an early stage, the rate of survival is significantly higher. When looking for liver tumours, CT is one of the most important and effective imaging methods [3,4]. Furthermore, CT provides entire liver pictures by contrast media injection and “multi-phase sequential scans” [5]. Due to the complexity of the CT image, manual segmentation adds significant time to the clinical workflow.

Segmentation is a crucial step in image processing and is also one of the more complex procedures involved in this field [6,7]. Automatically separating the tumour location from the liver is difficult due to factors including the liver’s varying size and shape among patients. In addition, when compared to other linked organs, such as the stomach and spleen, the liver’s intensity appears to be consistent throughout both high and low-contrast images [8,9]. In addition, images with low contrast and an ambiguous lesion shape make automatic segmentation of liver cancers challenging [10,11]. Issues with high susceptibility to noisy outliers and over and under-segmentation can plague several image segmentation methodologies such as active contrast segmentation, traditional segmentation technique, watershed model, and region expansion, resulting in less accurate results in less time [12,13].

**Figure 1:**
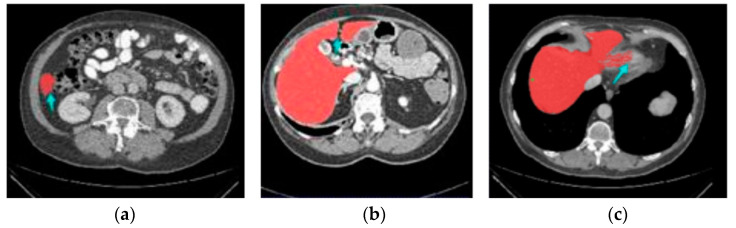
Three stimulating cases, including (**a**) Small liver zone, (**b**) Break liver part, (**c**) Blurred liver border.

Because certain lesions, such as hemangioma and metastasis, look similar to the liver, manual segmentation and organization of liver cuts from CT imageries is a lengthy task, leading to confusion and less reliable results [14]. Consequently, there is an urgent requirement for research into automated methods to aid radiotherapists in the diagnosis of liver scratches from CT scans [15]. Figure 1 demonstrates how difficult it is to detect liver regions with CT.

Researchers have created a wide variety of sophisticated methods in recent decades for autonomous liver segmentation. These methods may be loosely categorized into three groups: intensity-based approaches, machine learning-based approaches, and deep learning-based approaches. As a class, intensity-based tactics are known for their speedy execution, and this is especially true of thresholding-based approaches [16,17], district growth methods, and level-set methods. Most of these methods, however, are only semi-automatic, leaving them vulnerable to noise and requiring human involvement with complex stricture situations.

They allow for substantial gains in segmentation accuracy when used with ML-based approaches [18,19,20]. Most machine learning (ML)-based devices, however, necessitate the manual construction of specialized features, which significantly affects precision. CNN and other deep learning-based methods have seen tremendous growth because of their sophisticated subsequent triumphs in a variety of areas, including target identification, picture segmentation, and classification [21,22,23]. A Fully Convolutional Network (FCN) is a popular deep learning-based technique that performs exceptionally well at classifying images down to the pixel level. The accuracy of the deep learning-based methods is significantly higher than that of existing ML-based methods [24]. There are, however, constraints on both FCN and U-Net-based approaches. Both single network training and cascade structure training employing the FCN-based method have a high failure rate when it comes to producing reliable outcomes in the liver [17,25,26]. The fundamental reason for this is that when evaluating the link among pixels, a reduction in the capacity to notice subtle visual cues occurs [27]. While the U-Net-based method is effective, it refined the feature map only after the U-Net convolution process was completed. Further, as the network’s depth increases, the gradient vanishment problem becomes more easily triggered, and the picture resolution rapidly decreases due to the network’s constant down-sampling operation, which would negatively affect the regions [28]. Finally, the category imbalance problem might lead to mistakes in areas and unclear liver boundaries. While a 3D network’s learning effect would improve along with the z-axis information, in practice, this is difficult to achieve due to memory constraints, making the choice of the slice number tricky [29].

In most cases requiring automatic liver segmentation, the aforementioned strategies perform admirably. However, their accuracy and resilience remain insufficient when applied directly to clinical data, which severely limits their further use. The major contribution of the research work is as mentioned below:

- Three steps are only included in this study such as pre-processing, segmentation, and classification.
- Histogram Equalization and medium filtering are used for the improvement of the input images.
- Enhanced Mask R-CNN is used to segment the liver tumor from the pre-processed images. The research work introduces multistage optimization for deep learning segmentation networks. The study used a multi-optimization training system by utilizing stochastic gradient descent and adaptive moment estimation (Adam) with preprocessed CT images in enhanced Mask-RCNN.
- APESTNet is introduced in this study for classification. Overfitting issues in the Swin Transformer model are prevented by introducing Adversarial propagation in the classifier.

The following paper is constructed as follows: Section 2 discusses the related works of liver segmentation and classification. Section 3 presents a brief explanation of the proposed model. The validation analysis of proposed segmentation and classification with existing techniques are given in Section 4. Finally, the limitations and scientific contributions of the work are described in Section 5 and Section 6.

## 2. Related Works

Rela et al. [30] use an optimization-driven segmentation and classification perfect for trying to apply a unique approach to analyzing and categorizing liver tumours. Five stages, (a) pre-processing, (b) liver segmentation, (c) tumour segmentation, (d) feature extraction, and (e) classification, are involved in the generated model’s execution of the task. The acquired CT images are pre-processed in three steps, including contrast enhancement via histogram equalization and noise filtering via the median filter. In the next step after image preprocessing, CT abdominal images are segmented to isolate the liver using adaptive thresholding to train the classifier using the tumour picture segmentation. Two deep learning techniques, RNN and CNN, are utilized in the classification process. The CNN receives the segmented image of the tumour, while the RNN receives the extracted features. To further enhance the hidden neuron optimization, a hybrid classifier that has been further refined is utilized. In addition, an enhanced meta-heuristic approach.

Liver area extraction from CT scan images was proposed by Ahmad et al. [31] using a very lightweight CNN. In order to distinguish the liver from the background, the proposed CNN algorithm employs softmax across its three and two fully connected layers. Using a random Gaussian distribution to seed weights, we were able to embed these data while maintaining their semantic distances. Ga-CNN is the name of the proposed network (Gaussian-weight initialization of CNN). The MICCAI SLiver’07, 3 Dircadb01, and LiTS17 benchmark datasets are used in the experiments. Across all benchmark datasets, experimental results demonstrate the superiority of the suggested technique.

Using generative adversarial networks (GANs) and Mask R-CNN, Wei et al. [32] presented a technique for segmenting liver images. To begin, Mask R-CNN and GANs were investigated further to improve pixel-wise classification, as most output images contain noisy characteristics. To improve the segmentation performance, k-means clustering was then utilized to lock the image aspect ratio and obtain additional crucial anchors. Finally, we developed a GAN Mask R-CNN method, which outperformed state-of-the-art alternatives and the Multi-Image Classification and Analysis Improvement (MICCAI) measures. The suggested approach also outperformed ten state-of-the-art algorithms on six Boolean indications.

With its roots in the traditional U-Net, Wang et al. [33] presented a novel network design dubbed SAR-U-Net. After each convolution in the U-Net encoder, a SE block is presented to adaptively extract, hiding unimportant parts of the image and emphasizing parts that are essential to the segmentation task at hand. Second, the ASPP is used to acquire picture data at several scales and receptive fields by substituting the transition layer and the output layer. Third, the typical convolution block is swapped out for the residual structures to help with the gradient vanishment problem, and this causes the network to improve accuracy from a much higher depth. Five widely-used measures, including the Dice coefficient, VOE, RVD, ASD, and MSD, were employed in the LiTS17 database test. The proposed technique was the most accurate when compared to other similar models.

In this study, Roy et al. [34] present a novel automatic method for segmenting and classifying liver tumours. For classification purposes, the model employed a hybrid deep learning-based Convolution Neural Network (HCNN) model hybrid. The classification approach computes a multiclass categorization of the tumours discovered, while the segmentation framework seeks to distinguish between normal and malignant liver tissue. The focus of this study is on developing a method that eliminates the possibility of human mistakes in the forecasting process. On the other hand, the suggested method has recall values that are nearly as high as the best existing methods, and it delivers the highest precision for lesion identification. On average, the suggested method properly categorizes tumours in the liver as either hepatocellular carcinomas (HCC), malignant tumours other than HCC, or benign tumours or cysts. This paper’s novelties lie in its implementation of MSER to segment tumour lesions and its use of a hybrid CNN-based technique to classify liver masses.

Hussain et al. [35] zeroes in on the Machine Learning (ML) techniques of multiclass liver tumour classification using Random Forest (RF). There are four types of tumours included in the dataset: hemangioma, cyst, hepatocellular carcinoma, and metastasis. The photos were gray-scaled, and the contrast was enhanced using histogram equalization. The Gabor filter was used to lessen the amount of background noise, and an image sharpening technique was used to enhance what was already there. Employing texture, binary, histogram, and rotational, scalability, and translational (RST) methods, we were able to collect 55 features for each ROI, despite their varying pixel sizes. Twenty optimal features for classification were extracted from the original set of 55 using the correlation-based feature selection (CFS) method. The outcomes demonstrated that RF and RT were more accurate (97.48% and 97.08%, respectively) than J48 and LMT. A more accurate diagnosis of liver cancers will be possible with the aid of the revolutionary framework provided.

In order to categorize multi-organ 3D CT cancer, Kaur et al. [36] used a convolutional neural network. The suggested method has been validated using a dataset consisting of 63503 CT scans of patients with liver cancer acquired from The Cancer Imaging Archive (TCIA). This strategy uses a convolutional neural network (CNN) to classify CT pictures of liver cancer. Results for the classification have been calculated. When the data-enhanced volume slices, the validation accuracy increases to 99.1% from the original volume slices’ accuracy of 98.7%. When compared to other volume slices, the test accuracy of the data-augmented volume slice dataset is 93.1% higher on average. The primary benefit of this effort will be in assisting the radiation therapist in narrowing their attention to a specific region of the CT images.

Jeong et al. [37] offer an automated approach to segmenting the liver in CT scans and estimating its volume using a deep learning-based segmentation system. The framework was trained using data from 191 donors, and it showed promising results in four different segmentation tasks: for the left lobe (0.789), the right lobe (0.869), the caudate lobe (0.955), and the overall liver (0.899). Moreover, the R2 value for the volume estimate task was as high as 0.980, 0.996, 0.953, and 0.996. The outcomes proved that this strategy delivers precise and quantifiable liver segmentation outcomes, lowering the margin of error in liver volume estimation.

Militello et al. [38] generate and validate a radiomic model with radiographic features extracted from breast dynamic contrast-enhanced magnetic resonance imaging (DCE-MRI) from a 1.5 T scanner. Images were acquired using an eight-channel breast coil in the axial plane. The rationale behind this study is to demonstrate the feasibility of a radiomics-driven model that can be integrated into clinical practice using only standard-of-care DCE-MRI with the goal of reducing required image pre-processing.

The existing DL models didn’t focus on overfitting issues and generally used classification techniques for classification and segmentation. This will decrease the classification accuracy, and therefore, this research work focused on addressing the overfitting issue by adding Adversarial Propagation to the Swin transformer model.

## 3. Materials and Methods

Radiologists currently perform a slice-by-slice examination of numerous CT scans to segment liver tumours manually. Manual methods are more difficult and time-consuming. Computer-assisted diagnosis relies on the segmented regions, and human segmentation of images may compromise diagnostic accuracy. The main challenges of automatic liver and liver tumour segmentation models are (a) low contrast between the liver tumour and healthy tissue in CT images, (b) variable size, location, and shape of liver tumours, making segmentation difficult; and (c) the liver is closely connected with the adjacent organs, and the CT value of these organs will be similar to the livers.

“(a) Image pre-processing, (b) Liver and tumor segmentation, and (c) Classification” are the three steps that make up the suggested liver tumour segmentation and classification method. The CT images are first collected, then pre-processing techniques, such as histogram equalization and median filtering are applied to them. Histogram equalization is used to increase contrast, and median filtering is used to reduce noise in the final image.

### 3.1. Dataset and Implementation

The experiment makes use of the labeled training sets from the LiTS17 2 and SLiver073 datasets. There is a wide range of sampling techniques included in the 131–3 D abdominal CT scan sets that make up the LiTS17-Training dataset. With an in-plane resolution of 0.55 mm × 1.0 mm and inter-slice spacing of 0.45 mm × 6.0 mm, CT images and labels have a 512 × 512 pixel size. From a total of 131 datasets, 121 were chosen at random for use in our experiment’s training phase, while the remaining 10 were employed in the experiment’s testing phase. Additionally, all 20 datasets from the SLiver07-Training dataset were used for evaluation. Each CT image in this dataset is 512 × 512 pixels in size.

During training, the learning rate (lr) starts at 0.001 and decreases by 0.005 per four training iterations using the Formula (1):

lr = initial lr (epoch/step scope)(1)

where early lr and step size are both constants. For this purpose, the tried-and-true stochastic gradient descent (SGD) algorithm was employed.

It was empirically determined that a batch size of 4 was optimal for running the suggested approach on our GPU with 11 GB of memory. Epoch is empirically chosen to 60 to guarantee efficient training, as this is the point around which the majority of the training converges. All the tests are conducted on a workstation equipped with Ubuntu (Canonical Ltd., London, UK).

### 3.2. Image Pre-Processing

The histogram equalization and median filtering method are used for preparing the raw CT abdominal picture.

#### Histogram Equalization

It’s used as a preliminary processing step since it adjusts an image’s brightness to boost its contrast. Let $In^{im}$ be the input picture and define pixel value as the matrix of integer pixel intensities between 0 and 1. The number of intensity values is represented by INV, with a maximum value of 256 being the norm. Equation notation for the normalized histogram NHS of InHE with a bin to possible intensity (2). In this case, HE=0, 1,…, (INV−1). The equation for the histogram-normalized image is (3) [39]:

(2) $$NHS=\frac{Number\;of\;pixels\;with\;density\;he}{Total\;number\;of\;pixels}$$

(3) $$In^{HE}=floor\left(\left(INV-1\right)\overset{In_{\left(i,Q\right)}^{im}}{\underset{he=0}{\sum }}NHS\right)$$

The term floor() in the aforementioned equation rounds down to the next integer value. Thus, a median filter is applied to further smooth out the histogram-equalized image $In^{HE}$. Filtering data by taking the middle value [39]: Restricting low- and high-frequency pixels, as this filter does, removes noise from an image, making it easier to see and appreciate its edges. If you want to eliminate the noise in your liver image, try using a non-linear filter, such as median filtering. This filter’s primary function is to replace noisy pixels with the image’s median pixel value, which is calculated by ranking each pixel according to its grayscale value. When the median filter is applied to the input image, HE, we achieved the resulting image MF, as shown in Equation (4).

(4) $$In^{MF}\left(x,y\right)=med\left\{In^{HE}\left(x-u,y-v\right)u,v\in H\right\}$$

The original image and the median filtered image are represented by $In^{HE}$ and $In^{MF}$, respectively, in Equation (4). As an added bonus, H represents a 2-dimensional mask. Since this is the last step in the pre-processing phase, the resulting picture, $In^{MF}$, is next processed using liver segmentation.

### 3.3. Segmentation Using Enhanced M-RCNN

**Figure 2:**
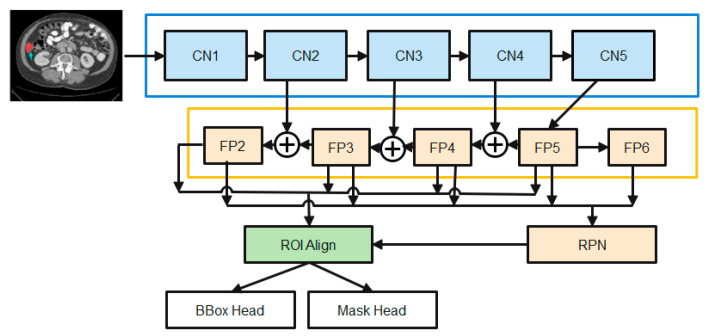
The diagrams of the Enhanced M-RCNN Framework.

The state-of-the-art in instance picture segmentation is the mask-RCNN framework, which the proposed method builds on. This framework has shown outstanding performance in a number of image segmentation studies. Figure 2 depicts the major steps that make up the proposed enhanced M-RCNN method: Backbone, Neck, DenseHead, and ROIHead (Region of Interest Head).

(1) The Backbone converts the incoming image into a raw feature map. Here, we employ a variant of ResNet-50-based on the design.
(2) The Neck joins the Spine to the Head. The original feature map is refined and rearranged. It has a vertical corridor and horizontal branches. To produce a feature pyramid map of the same size as the raw feature map, the top-bottom route is used. Convolutional add operations between two parallel pathways’ corresponding levels characterize lateral linkages.
(3) Third, the Dense Head can be used to perform dense placements of feature maps. The RPN examines each area and makes the best guess as to whether or not an object is present. The RPN’s main benefit is that it doesn’t need to look at the real image itself. Through the use of a fixed number of anchor boxes, the network performs a rapid scan of the feature map.
(4) ROIHead (BBoxHead, MaskHead): Using ROIAlign, extract features that affect the ROI from different feature maps while maintaining their precise spatial placements. This section takes in ROI features and then predicts task-related outcomes based on those features. At this point, you’ll be doing two things at once:
  - In the detection branch, the location of the bounding box (BBoxHead) is identified and classified for intervertebral disc (IVD) detection.
  - In the segmentation node, the FCN created the IVD image segmentation. b. MaskHead.

Classification loss and mask loss are all summed together unweighted to form the loss function. SGD optimization and the Adam optimization approaches are employed by an enhanced M-RCNN. Training makes use of SGD and Adam optimization. To identify global optimums, the SGD is effective and simple to utilize. When trying to find a local optimum, SGD fails and becomes difficult to employ. When it comes to optimizing sparse gradients in noisy situations, Adam optimization combines the best features of the adaptive gradient and root mean square propagation to create an effective technique.

At its core, our feature extractor is a ResNet-50 that has been initialized using ImageNet-trained weights. Xavier initialization is used for all other weights (such as the RPN). To accomplish our tasks, a system with a single graphics processing unit is employed. The initial mask-RCNN used a batch size of 16. There are three distinct stages to the training process, the first of which involves simply training the MaskHead and not the projected ResNet-50 backbone. Parts of the backbone [beginning at layer 4 (CN4)] and the prediction heads (DenseHead and ROIHead) are fine-tuned in the second stage. The third and final stage involves joint training of the model’s constituent parts (the “heads” and the “backbone”). The study employs liver image data with SGD optimization with Adam optimization during the third and final stage. Training is slowed to a crawl by setting alpha = 1.0 E-6 (learning rate or step size), beta1 = 0.9, beta2 = 0.999, and epsilon = 1 e−08.

Starting with the smallest feature map and working our way down to larger ones via upscale operations, the study employs this top-bottom strategy to build final feature maps. A feature map is produced in layer two of the diagram, and its use of 1 × 1 convolutions reduces the total number of channels to 256. The up-sampled output from the previous cycle is then combined with these components. The outputs of this procedure are fed into a 33-convolution layer with stride 2 to generate the last four feature maps (FP2, FP3, FP4, and FP5). Max-pooling from FP5 yields FP6, which is considered the fifth feature map. Using these five feature maps, RPN may create candidate object bounding boxes. However, when associating with ROIs, only four feature maps (FP2, FP3, FP4, and FP5) are used.

A series of convolutional layers, followed by batch normalization and the ReLU activation function, are standard fare in the original ResNet-50 architecture’s convolutional blocks and identity blocks. To better deal with training data that is jumbled together, the study employs group normalization and dropout regularization and makes many tweaks to these methods. The enhanced M-RCNN makes use of high-definition training data. Due to this, the study can only process a maximum of two photos at a time in a batch. While a small batch size can lead to an inaccurate estimation of the batch statistics, a big batch size is necessary for effective batch normalization. The model error may increase dramatically if the batch size is decreased. In this case, dropout regularization is applied after group normalization. To avoid model overfitting and boost the generalization effect, regularization is a must in deep learning. To eliminate co-adaptation issues among the hidden nodes of deep feedforward neural networks, the dropout regularization strategy has been effectively used in several deep learning models.

#### Loss Functions

In enhanced M-RCNN, the loss is computed as a total of losses at each stage of the model. The cost represents the weights that each stage of the model should have. For classification and bounding box regression, the ROIAlign output is fed into the BBo × Head, while for segmentation, the output is fed into the MaskHead. The output of the FCN layer is sent into a softmax layer, which performs the classification utilizing all of the characteristics. The MOM-RCNN loss function demonstrates the deviation between the predicted and observed values. As a result, the study presents a single loss function for training the bounded RCNN’s box refinement regression, class prediction classification, and mask prediction generation. Mask prediction generation loss (*L_mask_*) is only obtained from mask prediction generation stages, while class prediction classification loss $L_{r,class}$ and $L_{m,class}$ and bounding box refinement regression loss are obtained from both the RPN and mask prediction generation stages. It is only necessary to specify the L mask once per class, which eliminates output competition amongst masks. The resulting enhanced M-RCNN loss function is defined as (5):

(5) $$L_{enhacned\;M-RCNN}=L_{r,class}+L_{m,class}+L_{r,box}+L_{m,box}+L_{mask}$$

where $L_{r,class}$: This is the monetary cost associated with an RPN’s mistaken identification of anchor boxes (the presence/absence of an object). In cases where the final output model is not picking up on many objects, this value should be high so that RPN can record it. $L_{r,box}$: What this means is that the RPN is quite precise in its localization. When the object is recognized, but its bounding box needs adjusting, this is what you utilize to fine-tune the detection. $L_{m,class}$: This is the cost associated with misidentifying an item in the designated area. In the likely scenario where the object is recognized from the image but incorrectly labeled, this probability is high. $L_{m,box}$: To put it another way, this is the same as the “loss” calculated when pinpointing the precise location of the boundary of the specifically named class. It’s high if the object is properly classified, but localization is off. $L_{mask}$: Masks were made based on the things that were detected. Therefore, this is related.

The class prediction organization error ($L_{r,class}$ and $L_{m,class}$) is calculated by (6):

(6) $$L_{r,class}=\frac{1}{M_{class}}\sum_{i}-\log\left[pr_{i}^{*}pr_{i}+\left(1-pr_{i}^{*}\right)\left(1-pr_{i}\right)\right]$$

where MM class is the total number of classes and $pr_{i}$ is the likelihood that the ith region of interest (ROI) contains a positive sample (liver). If the ROIs are comprised of positive samples, then =1, and else it will be 0. $L_{m,class}$ follows the same formula.

Regression loss $L_{r,class}$ and $L_{m,class}$ are calculated by plugging these two values into an Equation (7):

(7) $$L_{r,box}=\frac{1}{M_{regress}}\underset{i}{\sum }pr_{i}^{*}S\left(trans_{i},trans_{i}^{*}\right)$$

*S*() is a smooth function where *M* regress is the sum of pixels in the feature map, $trans_{i}$ shows $L_{m,box}$ follows the same formula. The loss *L* mask in mask prediction generation is calculated by (8):

(8) $$L_{mask}=-\frac{1}{n^{2}}\underset{1\leq x,y\leq n}{\sum }\left[lbl_{xy}^{p}=\left(1-lbl_{xy}\right)\log\left(1-lbl_{xy}^{2}\right)\right]$$

where the label value at position (*x*, *y*) is denoted by $lbl_{xy}$ in the *n* by *n* region, and the predicted value for the *p*-th class is denoted by $lbl_{xy}^{p}$.

### 3.4. Classification

Though the transformer was first developed for processing natural language sequences, its application to CNN for image processing is to consider the picture as a matrix for convolution operation. Unfortunately, CNN is not well-suited for direct usage in picture feature extraction. That’s why patching techniques, such as patch embedding, patch merging, and masking are used.

#### 3.4.1. Patch Embedding

Patch partition is used to divide an RGB map into individual patches that do not overlap. Here, the size of the patch is 4 × 4, which is multiplied by the RGB channels to yield a total size, i.e., 4 × 4 × 3 = 48. To generate a feature matrix, we simply project the refined patchwork to the required dimensions.

#### 3.4.2. Patch Merging

After partitioning the obtained feature matrix into four 22 windows and merging their respective positions, the resulting four feature matrices are concatenated.

#### 3.4.3. Mask

When the pixels are subsequently relocated, the mask will only allow the window to focus on the continuous portion, reducing the influence of ingesting. If you shift the matrix to the right, the original window in the bottom right corner will be found. The size of the shift is proportional to window size and may be calculated using the following formula.

(9) $$s=\left[\frac{w}{2}\right]$$

for each given shift *s*, the window width *w* must be specified.

Because the region visible in the window to the right and below does not border the section in the original matrix, it must be separated from it using the mask matrix. Both the vertical and horizontal slicing areas are [0,-window size),[-window size,-shift size),[-shift size]. The concept behind the window partition (function window partition) for the labeled mask matrix is to equally split the window size into blocks of [H/w] rows [H/w] columns and combine the dimensions for the number and the batch size. The original matrix mask will be subdivided into smaller windows so that they can be individually counted as window units.

**Figure 3:**
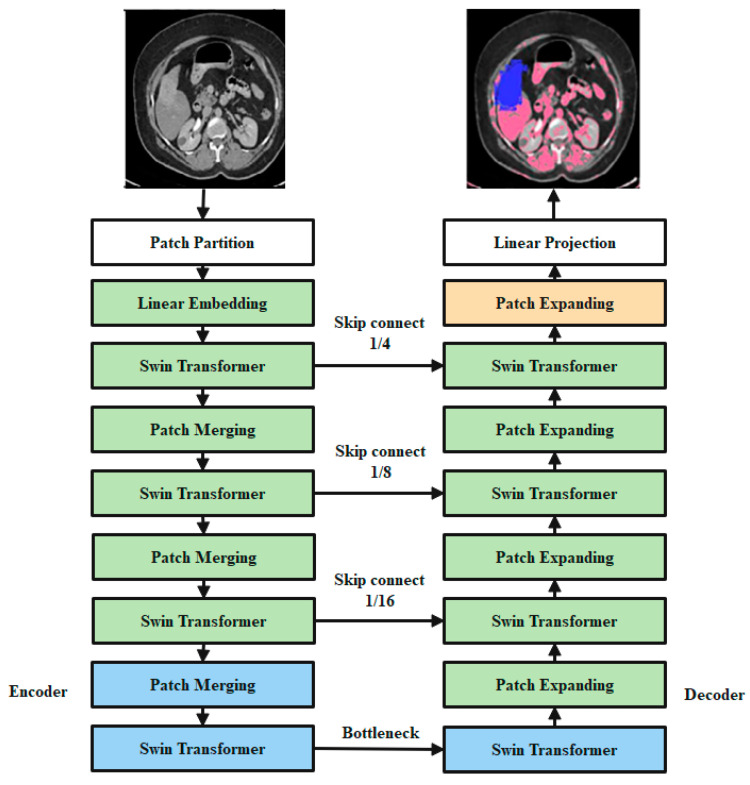
Encoders, bottleneck nodes, decoders, and skip links make up Swin-overall Unet’s structure. The swin transformer block is the foundation of the encoder, bottleneck, and decoder.

In Figure 3, the overall design of the proposed SwinNet is seen. Encoder, bottleneck, decoder, and skip links make up SwinNet. Swin Transformer blocks are Swin-fundamental Unet’s building block [40]. The medical images are divided into 4 × 4 non-overlapping patches for the encoder to use in transforming the inputs into sequence embeddings. After applying this partitioning strategy, the feature dimension of each patch is 4 × 4 × 3 = 48. Furthermore, the dimensions of the projected features are embedded into an arbitrary dimension using a linear embedding layer (represented as C). To create the hierarchical feature representations, the modified patch tokens are fed into multiple Swin Transformer blocks and patch merging layers. When it comes to downsampling and dimension expansion, the patch merging layer is in charge, whereas the Swin Transformer block is in charge of feature representation learning. We created a symmetric transformer-based decoder after being inspired by U-Net [24]. The decoder uses a Swin Transformer block and a patch-expanding layer. To compensate for the reduction in spatial detail brought on by down-sampling, the encoder’s multiscale features are fused with the retrieved context features via skip connections. A patch expanding layer, as opposed to a patch merging layer, is purpose-built for up-sampling. Through an up-sampling ratio of 2, the patch expanding layer converts 2D feature maps into 4D feature maps. The segmentation predictions at the pixel level are generated by applying a linear projection layer to the up-sampled features, and the input resolution of the feature maps is restored via a 4 up-sampling operation carried out by the final patch expanding layer. Our breakdown of the blocks’ descriptions would go as follows:

#### 3.4.4. Swin Transformer Block

**Figure 4:**
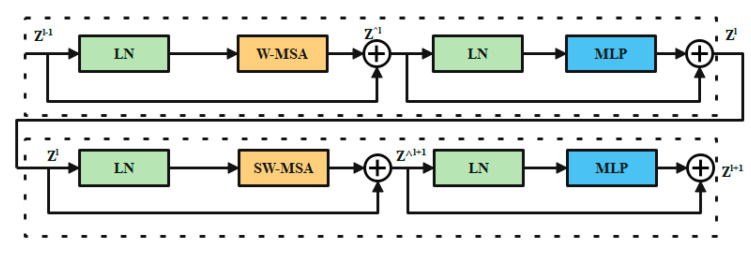
Swin transformer chunk.

In place of the standard multi-head self-attention (MSA) module, the swin transformer block [41] is constructed using shifted windows. Identical transformer pairs, as depicted in Figure 4. Each swin transformer block consists of LayerNorm (LN) layers, multi-head self-attention modules, residual connections, and two-layer MLPs with GELU non-linearity. A shifted window-based multi-head self-attention (SW-MSA) module is used in the first transformer block, while a window-based multi-head self-attention (W-MSA) module is used in the second. Continuous swin transformer blocks are created using this type of window partitioning (10)–(13):

(10) $${\hat{z}}^{l}=W-MSA\left(LN\left(z^{l-1}\right)\right)+z^{l-1}$$

(11) $$z^{l}=MLP\left(LN\left({\hat{z}}^{l}\right)\right)+{\hat{z}}^{l}$$

(12) $${\hat{z}}^{l+1}=SW-MSA\left(LN\left(z^{l}\right)\right)+z^{l}$$

(13) $$z^{l+1}=MLP\left(LN\left({\hat{z}}^{l+1}\right)\right)+{\hat{z}}^{l+1}$$

where ${\hat{z}}^{l}$ and $z^{l}$ stand for the results produced by the lth block’s SW-MSA module and the lth block’s MLP module, respectively. Self-attention is calculated in the same way as in earlier publications [42,43] (14):

(14) $$Atention\left(Q,K,V\right)=SoftMax\left(\frac{QK^{T}}{\sqrt{d}}+B\right)V$$

where *Q*, *K*, and *V* are the matrices in the space R(M2 d). The sum of patches in a window, M2, and the measurement of the query, d, are both variables. Furthermore, *B* is populated with numbers derived from the bias matrix B=R((2M−1)(2M+1)).

#### 3.4.5. Encoder

Tokenized inputs in C-dimensions at a resolution of H/4 W/4 are sent into two successive Swin Transformer blocks in the encoder for representation learning with the same feature dimension and resolution. At the same time, the patch merging layer will double the feature dimension while halving the token count. In the encoder, this process will be performed three times.

Integration layer for patches: The patch merging layer takes the input patches and combines the four subsets into one. This type of processing will result in a 2× down sampling of feature resolution. Moreover, a linear layer is applied to the concatenated features in order to reduce the feature dimension by a factor of four, making it equal to the original size of two.

#### 3.4.6. Bottleneck

Since Transformer cannot be converged [44], the bottleneck utilized to learn the deep feature representation is made up of just two successive Swin Transformer blocks. Both the feature size and resolution are maintained in the bottleneck.

#### 3.4.7. Decoder

The symmetric decoder is constructed using the same Swin Transformer block that was used in the encoder. Due to this, we up-sample the deep features collected by the decoder using the patch expanding layer rather than the patch merging layer. When the feature dimension is doubled.

Flare-up Expanding Layer: In order to raise the feature dimension from its initial value of (W/32 H/328 C) by 2 prior to up-sampling the features. The input characteristics are then rearrange-operated to increase their resolution by a factor of two and decrease their dimension by a factor of four (from W/32 H/3216 C to W/16 H/164 C).

#### 3.4.8. Skip Connection

To combine the encoder’s multi-scale characteristics with the up-sampled features, we use skip connections, similar to how the U-Net does. As a means of mitigating the loss of spatial information brought on by down-sampling, we join together both shallow and deep features. After an up-sampling layer, a linear layer is applied, maintaining the same dimensionality of the concatenated features as the up-sampled features. In this network, overfitting is a major issue that must be resolved to attain high classification accuracy. For this issue, adversarial propagation (AdvProp) [45] is used as an improved training scheme, which is used as a separate auxiliary batch norm for the training samples.

## 4. Results and Discussion

### 4.1. Segmentation Results

#### Evaluation Metrics for Segmentation

Dice coefficient (DC), Volume overlap error (VOE), Relative volume error (RVD), Average symmetric surface distance (ASD), and maximum surface distance (MSD) are the five most widely used metrics for assessing liver segmentation (MSD). The meanings of the five metrics are as follows, with A being the liver segmentation result and B representing the ground truth:

Dice coefficient (DC): the resemblance between two sets, with a range of (0,1). Greater values indicate more precise segmentation (15).

(15) $$Dice\left(A,B\right)=\frac{2\left|A\cap B\right|}{\left|A\right|+\left|B\right|}$$

Volume Overlap Error (VOE): volumetric discrepancy between segmented and raw data (16).

(16) $$VOE=1-\frac{\left|A\cap B\right|}{\left|A\cap B\right|}$$

RVD: Whether the result is over-segmented is measured with this metric. Values closer to 0 indicate more precise segmentation (17)–(19).

(17) $$RVD\left(A,B\right)=\frac{\left|B\right|-\left|A\right|}{\left|A\right|}$$

(18) $$ASD\left(A,B\right)=\frac{1}{\left|S\left(A\right)\right|+\left|S\left(B\right)\right|}\left(\underset{p\epsilon S\left(A\right)}{\sum }d\left(p,S\left(B\right)\right)+\underset{q\in S\left(B\right)}{\sum }d\left(q,S\left(A\right)\right)\right)$$

(19) MSD(A,B)=max{dp∈S(A)max (p,S(B)),dq∈S(B)max (p,S(A))}

**Table 1:** The results of proposed segmentation technique on 10 LiTS17-Training datasets.

Case Num	VOE	ASD (mm)	MSD (mm)	RVD	Dice
1	0.103	1.568	28.453	−0.029	0.955
2	0.089	1.471	34.951	−0.033	0.963
3	0.100	1.382	31.259	−0.049	0.956
4	0.097	1.494	25.494	−0.048	0.968
5	0.114	1.797	28.315	−0.040	0.949
6	0.107	1.933	29.756	−0.038	0.953
7	0.094	1.229	30.657	0.034	0.960
8	0.073	0.955	39.421	0.043	0.961
9	0.086	1.673	34.598	0.036	0.954
10	0.090	1.863	28.534	0.040	0.952
**Avg**	**0.095**	**1.544**	**29.144**	**−0.0084**	**0.957**

The dice measure of proposed enhanced M-RCNN achieved 0.957 on average results, where the ASD is 1.544, 0.095 of VOE, 29.144 mm of MSD, and −0.0084 of RVD on average results. A better Dice coefficient means better segmentation accuracy for an efficient proposed model. Here, the proposed model achieved better Dice, which is proved in the above Table 1. Table 2 presents the comparative analysis of the proposed segmentation model with existing techniques on the second dataset.

In Table 2, the experiments represent the Quantitative comparison with methods on 20 Sliver07-Training datasets. Here we have used different evaluations for the proposed method. Based on the analysis, it is clearly proven that the proposed model achieved better results than existing techniques. In the next section, the proposed classifier’s validation is carried out, and the results are provided.

### 4.2. Classification Results

#### 4.2.1. Performance Measure for Classification

Our ideal and comparable baseline projections are evaluated using a wide range of indicators. An exhaustive list of evaluation criteria is provided below:

- Accuracy: On test samples, accuracy is referred to as “accuracy.”
- Precision: In the context of predictive value, precision refers to a positive value and is the ratio of genuine positive models to the total number of false positive samples.
- Recall: Classifier performance can be evaluated using this metric. Alternatively known as Sensitivity or True Positive Rate, which describes an organization model, Recall discards a positive prediction if it’s not accurate.
- F1: Classification is an example of a machine learning task for which this measure is well-known. It is the arithmetic mean of the estimates’ precision and recall.

#### 4.2.2. Validation Analysis Using 70% of Training Data–30% of Testing Data

In the above Table 3 represents the comparative Analysis of the Proposed Model. In these evaluations, the proposed model achieves better performance than other models, such as the proposed model accuracy of 95.62%, F-1 measure of 94.53%, precision of 98.32%, and recall of 94.62%, respectively. However, the existing techniques achieved nearly 92% to 94% accuracy, 93% to 96% precision, 89% to 92% recall, and 87% to 92% F1-measure.

#### 4.2.3. Validation Analysis Using 60% of Training Data–40% of Testing Data

**Figure 5:**
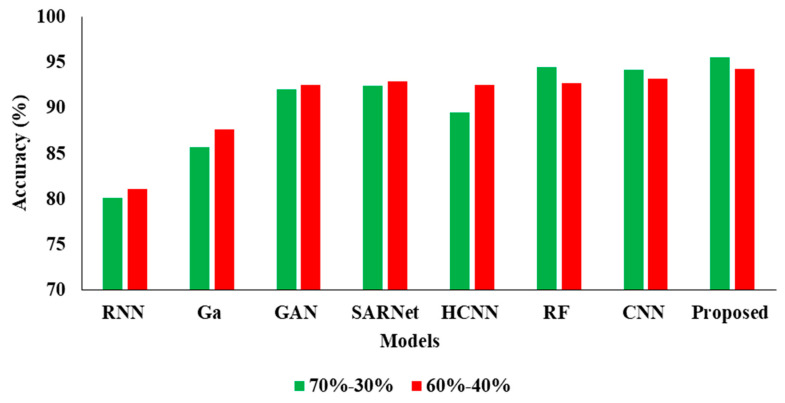
Accuracy Comparison for two data splits-ups.

**Figure 6:**
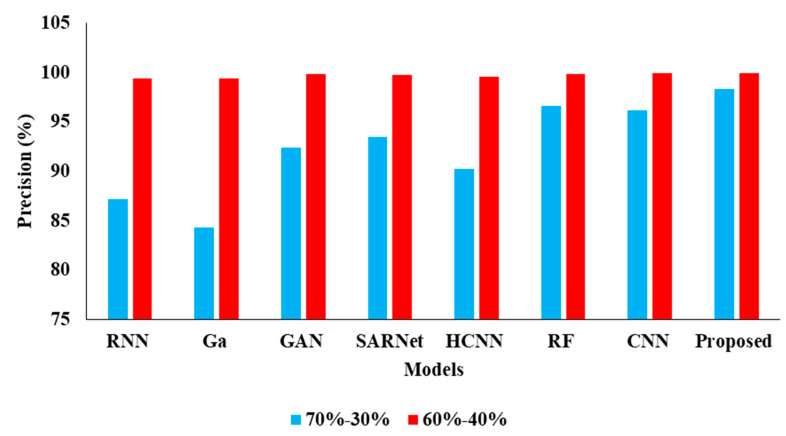
Precision Comparison for two data splits-ups.

**Figure 7:**
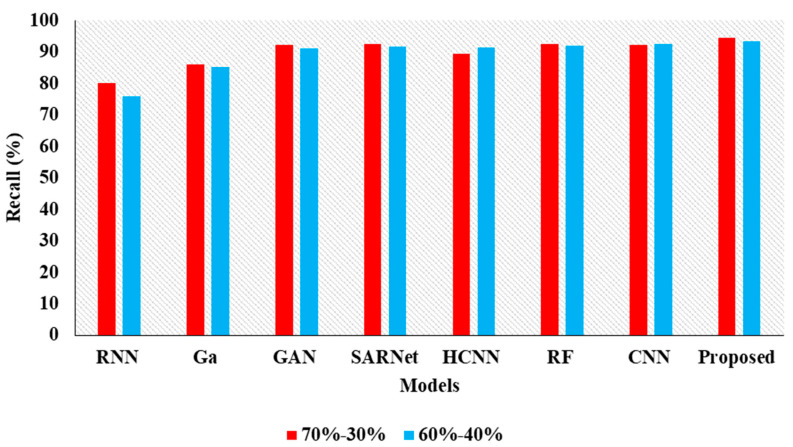
Recall Comparison for two data splits-ups.

**Figure 8:**
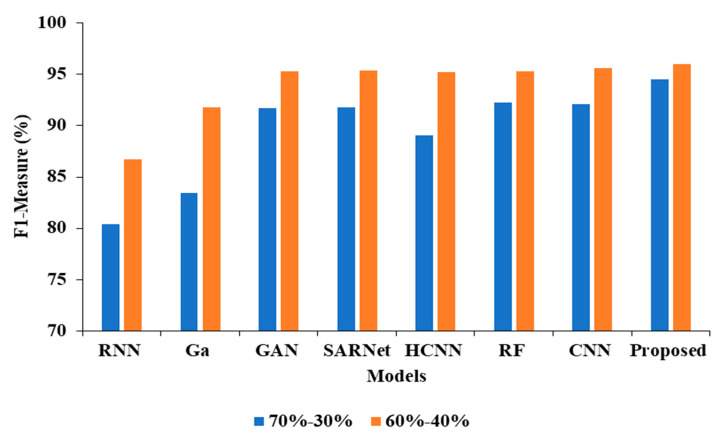
F1-measure Comparison for two data splits-ups.

In the Table 4, the results represent the Comparative Analysis of the Proposed Model. In these evaluations, the proposed model data is split into 60–40% percentages, and the performance achieves better performance than other models. For instance, the proposed model has an accuracy of 94.32% and a recall of 93.24%, respectively. Here, data plays a major role in the performance analysis, which is clearly proven in Figure 5, Figure 6, Figure 7 and Figure 8.

**Table 4:** Comparative Analysis of Proposed Model with existing techniques.

Model	Accuracy	Precision	Recall	F1
CNN-RNN [30]	81.10	99.41	75.91	86.72
Ga-CNN [31]	87.70	99.41	85.21	91.82
GAN-R-CNN [32]	92.50	99.82	90.98	95.27
SAR-U-Net [33]	92.90	99.78	91.52	95.41
HCNN [34]	92.50	99.63	91.38	95.18
RF [35]	92.70	99.90	91.93	95.32
CNN [36]	93.27	99.91	92.47	95.63
**Proposed APESTNet**	**94.32**	**99.95**	**93.24**	**96.02**

The Table 5 represents the Comparative Analysis of the Proposed Model. From these validations, it proves that the performance of the proposed model achieves better performance than other models. For instance, the proposed model training period is 390.33, and the execution time is 0.01128 (s), respectively.

## 5. Limitation

While the proposed methodology yielded some promising outcomes, there remains room for improvement. The CT pictures are 3D, but the suggested method is built on a 2D network; thus, it can easily lose crucial context information along the z-axis. In addition, the proposed technique may produce substantial mistakes around the boundary when the liver margin has lesions or tumour abnormalities.

## 6. Conclusions

This research introduces a novel model called APESTNet to improve the classification and categorization of liver tumours. The built-model consists of three phases: pre-processing, segmentation, and classification. The acquired CT images were subjected to histogram equalization and a median filtering technique before further analysis could be performed. After the necessary steps were taken to prepare the data, a tumour was segmented using an upgraded mask R-CNN model. The classification is then performed later by an interactively learning Swin Transformer block, the core unit for feature representation and long-range semantic information. In particular, the proposed strategy improved significantly and was very resilient while dealing with small liver pieces, discontinuous liver regions, and fuzzy liver boundaries. The experimental results confirm that the proposed APESTNet is more effective in classifying liver tumours than the current state-of-the-art models. Without compromising accuracy, the proposed method conserved resources. However, the proposed method is prone to slight over-segmentation or under-segmentation errors when dealing with lesions or tumours at the liver boundary. Therefore our future work will concentrate on completely utilizing the z-axis information in 3D to reduce errors.

## Figures and Tables

**Table 2:** The results of a quantitative comparison with approaches on 20 Sliver07-Training datasets.

Methods	Dice (%)	VOE (%)	RVD (%)	ASD (mm)	MSD (mm)
Adaptive Thresholding [30]	95.60 ± 3.41	8.23 ± 5.89	−2.38 ± 2.16	2.19 ± 0.38	36.69 ± 1.45
Ga-CNN [31]	96.94 ± 1.78	5.31 ± 3.48	−0.54 ± 2.24	1.95 ± 0.34	30.66 ± 2.03
GAN Mask R-CNN [32]	96.66 ± 2.19	6.38 ± 3.93	−1.29 ± 3.58	1.80 ± 0.38	28.30 ± 2.05
Proposed Enhanced M-RCNN model	**97.31 ± 1.49**	**5.37 ± 3.27**	**−1.08 ± 2.06**	**1.85 ± 0.30**	**27.45 ± 1.89**

**Table 3:** Comparative Analysis of Proposed Model.

Model	Accuracy	Precision	Recall	F1
CNN-RNN [30]	80.10	87.21	80.15	80.43
Ga-CNN [31]	85.71	84.32	85.93	83.45
GAN-R-CNN [32]	92.10	92.43	92.15	91.68
SAR-U-Net [33]	92.46	93.48	92.44	91.81
HCNN [34]	89.52	90.21	89.54	89.03
RF [35]	94.53	96.61	92.52	92.24
CNN [36]	94.16	96.17	92.32	92.10
**Proposed APESTNet**	**95.62**	**98.32**	**94.62**	**94.53**

**Table 5:** Comparison of the proposed model for different time executions.

Model	Training Time (s)	Testing Time (s)	Execution Time (s)
CNN-RNN [30]	630.01	69.17	0.01233
Ga-CNN [31]	450.53	67.38	0.01369
GAN-R-CNN [32]	543.21	65.89	0.01369
SAR-U-Net [33]	577.66	63.71	0.01657
CNN [34]	480.23	60.41	0. 01309
RF [35]	583.42	59.78	0. 01297
CNN [36]	423.17	59.192	0. 01289
**Proposed APESTNet**	**390.33**	**57.621**	0.01128

## Data Availability

The datasets used and/or analyzed during the current study are available from the corresponding author upon reasonable request.
